# The role and gene expression profile of SOCS3 in colorectal carcinoma

**DOI:** 10.18632/oncotarget.23477

**Published:** 2017-12-20

**Authors:** Xing Dong, Jing Wang, Bo Tang, Yeng-Xue Hao, Ping-Yang Li, Shi-Yong Li, Pei-Wu Yu

**Affiliations:** ^1^ Department of General Surgery, Southwest Hospital, Third Military Medical University, Chongqing, China; ^2^ Department of General Surgery, PLA Army General Hospital, Beijing, China; ^3^ Department of Obstetrics and Gynecology, PLA Army General Hospital, Beijing, China

**Keywords:** SOCS3, colorectal carcinoma, mechanisms, indicator, gene therapy

## Abstract

SOCS3 has been postulated to play a role in the occurrence and progression of malignancies. However, the relationship of SOCS3 with colorectal carcinoma remains poorly understood. The purpose of the study was to explore the role of SOCS3 in colorectal carcinoma and its underlying mechanisms. Protein and mRNA expression of SOCS3 in colorectal carcinoma and normal colorectal mucosa was detected using immunohistochemistry and real-time quantitative PCR. SOCS3 expression was significantly lower in colorectal carcinoma tissue than in normal colorectal mucosa, and was negatively correlated with tumor invasion depth, lymph node metastasis, differentiation degree, and TNM stage. A stably transfected colorectal carcinoma cell line (8348SOCS3) with high expression of SOCS3 was established. The effects of SOCS3 overexpression on the growth, proliferation, invasion and tumor formation of colorectal carcinoma cells were examined by CCK-8 assay, transwell method and tumorigenicity assays in nude mice. Then we found SOCS3 overexpression significantly decreased proliferation and invasion capability of 8348 cells *in vitro* and *in vivo*. Furthermore, the effect of SOCS3 overexpression on the gene expression profile of colorectal carcinoma cells was analyzed using human genome arrays. The results revealed 369 genes that were differentially expressed in 8348SOCS3 cells. 193 genes was significantly increased and 176 genes was significantly decreased. Bioinformatics analysis demonstrated that high SOCS3 expression affected multiple signaling pathways in colorectal carcinoma including TGF-β/Smads, NF-κB, and HIF-MAPK pathways. Especially for the TGF-β/Smads pathways, high SOCS3 expression could inhibit TGF-β1 expression and activate Smad4 expression. These data suggested that low expression of SOCS3 was associated with the occurrence and progression of colorectal carcinoma. SOCS3 protein may be a useful indicator for malignancy and prognosis of colorectal carcinoma and also a new target for gene therapy.

## INTRODUCTION

Colorectal carcinoma (CRC) is one of the most common clinical gastrointestinal tumors and its prevalence is increasing annually [1, 2]. The pathogenesis of CRC and its metastases remains unclear, and there is currently no effective treatment for this malignancy. Studies have confirmed that CRC is a multi-stage disorder caused by multiple factors and multiple genetic variations and involving the activation of oncogenes, inactivation of tumor suppressor genes, and disorders of apoptosis [3, 4]. The suppressor of cytokine signaling (SOCS) family plays a key role in regulating the growth and differentiation of cells. In particular, the role of the SOCS family in the pathogenesis of malignant tumors has increasingly been recognized [5–7]. As a key member of the SOCS family, SOCS3 participates in the regulation of multiple signaling pathways. In our previous study, we showed that gene silencing of SOCS3 by siRNA significantly improved the erythroid development of human hematopoietic stem cells [8]. Numerous studies have shown that SOCS3 expression can be induced by a variety of pro- and anti-inflammatory cytokines. It can suppresses the signaling of multiple immune molecules. Since it exerts key roles in the development of inflammatory diseases, viral infections, obesity and cancer, SOCS3 may be a biomolecular indicator for disease diagnosis and prognostic prediction. It is also serve as a potential target for the treatment of a variety of diseases [9, 10]. Research has shown that expression of SOCS-3 is decreased in many malignant tumor tissues and cell lines, such as head and neck squamous cell carcinoma, liver cancer, renal carcinoma, melanoma, and prostate cancer [11–13]. whereas demethylation or recovery of SOCS-3 expression via approaches such as drug treatment can significantly inhibit the growth of tumor cells, suggesting that SOCS-3 may act as a tumor suppressor gene.

To date, few studies have explored the role of SOCS3 in the invasion and metastasis of CRC and the underlying mechanisms. In the current study, we investigated the roles of SOCS3 protein in the pathogenesis of CRC at cellular and organismal levels and elucidated relevant regulatory signals and pathways. Our study revealed action mechanisms of SOCS3 and provided a new predictive indicator and potential therapeutic target for CRC.

## RESULTS

### Expression of SOCS3 in CRC and its significance

#### Expression of SOCS3 in CRC and normal colorectal tissue by immunohistochemical method

**Figure 1:**
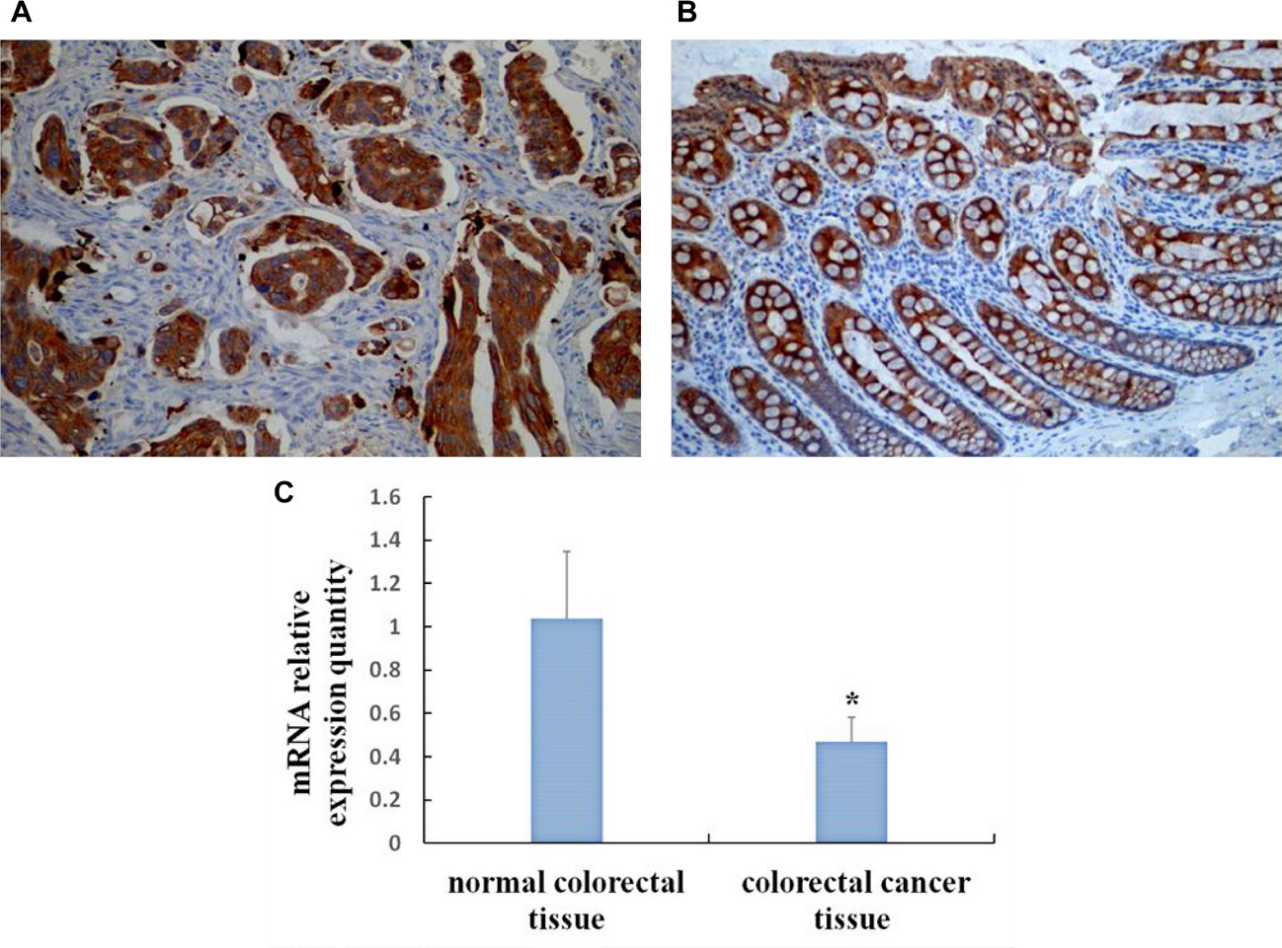
Expression of SOCS3 in CRC and normal colorectal tissue by immunohistochemical method and qRT-PCR (**A**) Positive expression of SOCS3 protein in Colorectal cancer tissues (SP, × 200); (**B**) Positive expression of SOCS3 protein in Normal colorectal tissues (SP, × 200); (**C**) SOCS3 mRNA levels in colorectal cancer tissue and normal colorectal tissue. ^*^P < 0.01, compared with normal colorectal tissue.

Positive SOCS3 expression was localized in the cytoplasm (Figure 1A–1B). The expression of SOCS3 in CRC tissue (32.5%, 13/40) was significantly lower than that in normal colorectal tissues (77.5%, 31/40) (χ^2^ = 16.364, *P* < 0.01). SOCS3 expression in CRC tissue was not correlated with gender, age, and tumor size (all *P* > 0.05), but was negatively correlated with invasion depth, lymph node metastasis, differentiation degree, and TNM stage (all *P* < 0.05) (Table 1).

**Table 1:** Relationship between SOCS3 expression and clinicopathological features of CRC

Clinicopathologic factors	*n*	SOCS3			
		+	-	χ^2^ value	*P*
Gender					
Men	28	8	20	0.195	> 0.05
Women	12	5	7		
Age (years)					
≤ 40	14	4	10	0.001	> 0.05
> 40	26	9	17		
Tumor size					
≤3 cm	27	7	20	0.845	> 0.05
*n* > 3 cm	13	6	7		
Differentiation degree					
Well/moderately differentiated	21	10	11	4.607	< 0.05
Poorly differentiated	19	3	16		
Depth of invasion					
Mucosa or superficial muscular layer	17	9	8	5.631	< 0.05
Deep muscular layer or whole layer	23	4	19		
Lymph node metastasis					
Yes	25	4	21	6.389	< 0.05
No	15	9	6		
TNM stage					
I+II	18	10	8	6.134	< 0.05
III+IV	22	3	19		

### Quantitative expression of SOCS3 in CRC and normal colorectal tissues by qRT-PCR

The expression of SOCS3 mRNA in CRC tissues (0.467 ± 0.113) was significantly lower than that in normal colorectal mucosa (1.035 ± 0.312) (t = 10.826, *P* < 0.01) (Figure 1C).

#### Effect of SOCS3 overexpression on the biological features of CRC cells

### Generation of CRC cell line with stable high SOCS3 expression

#### Packaging of lentivirus

**Figure 2:**
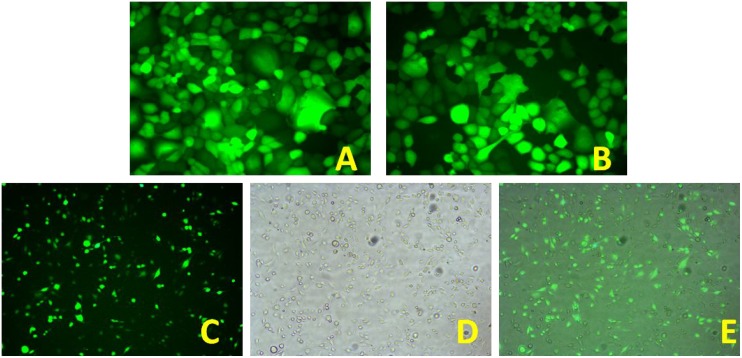
(**A**) 293 packaging cells that secrete lentivirus particles (× 40); (**B**) 293 cells observed under a fluorescence microscope (× 40). (**C**) GFP-positive 8348 cells after sorting (× 10); (**D**) 8348 cells under a light microscope after sorting (× 10); (**E**) Merged image of C and D.

During packaging of the 293 cells to produce lentivirus, the envelope glycoprotein could fuse the 293 cells; as a result, the formation of multinucleated syncytia could be seen under light microscopy (Figure 2A). Expression of GFP in 293 cells could be observed under a fluorescence microscope. As shown in Figures 2A and 2B, expression of GFP was observed in more than 95% of the 293 cells after 72 h.

### Transfection of lentivirus into 8348 cells and screening of positive cells

The 8348 cells were plated in 6-well plates. After cell density reached 70%, we added culture medium containing viral particles and an appropriate amount of polybrene and the cells were cultured at 37°C for 12–16 hours. After the viral supernatant was discarded, the cells were cultured in 8348 growth medium for another 48 h. Fluorescence microscopy showed that the percentage of 8348 cells with expression of GFP was less than 30%. To obtain cells that could stably express SOCS3 expression vector, flow cytometry was performed to sort the transfected 8348 cells based on the expression profile of GFP. As shown in Figure 2C–2E, after sorting the percentage of cells expressing GFP was greater than 90%.

### Comparison of SOCS3 expression in 8348SOCS3 cells and 8348plv cells by western blotting and qRT-PCR

**Figure 3:**
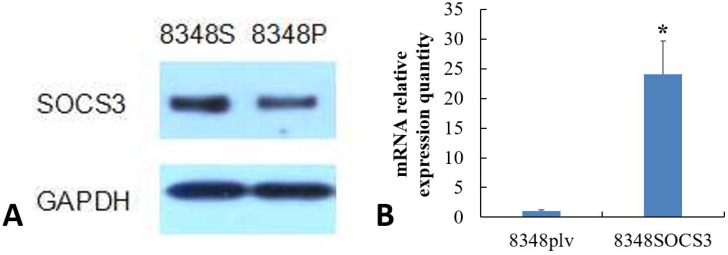
(**A**) SOCS3 protein expressions in 8348SOCS3 and 8348plv cells by western blotting. (**B**) SOCS3 mRNA expression in 8348SOCS3 and 8348plv cells by qRT-PCR. ^*^P < 0.01, compared with 8348plv cells.

The expression of SOCS3 mRNA and protein was significantly higher in 8348SOCS3 cells than that in 8348plv cells (Figures 3A–3B), confirming generation of a CRC cell line with stable high SOCS3 expression.

### Effect of high SOCS3 expression on the growth and proliferation of CRC cells by CCK-8 method

**Figure 4:**
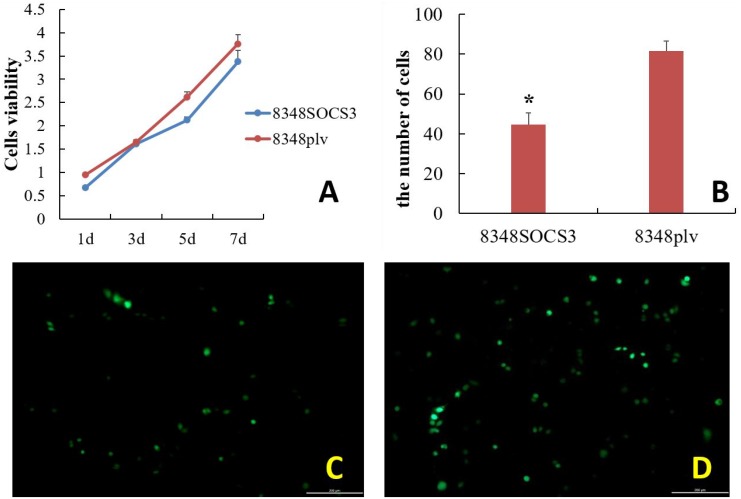
Effect of high SOCS3 expression on the growth, proliferation and invasion capability of CRC cells (**A**) Growth curve of colorectal cancer cells with and without transfection with SOCS3. (**B**) The number of cells penetrating the membrane was significantly lower in the 8348SOCS3 group than in the 8348plv group (^*^P < 0.05). Fluorescence microscopy of 8348 cells after culture in transwell chamber for 24 h. (**C**) 8348SOCS3 cells; (**D**) 8348plv cells.

8348SOCS3 cells showed a significantly slower proliferation rate than control cells. After transfection for 1, 5, and 7 days, there was a significant difference in the proliferation rate between the 8348SOCS3 group and 8348plv group (t = 10.654, t = 7.947, and t = 3.134, respectively; all *P* < 0.05) (Figure 4A).

### Effect of high SOCS3 expression on the invasion capability of CRC cells by transwell method

After routine culture of the cells in transwell plates for 24 h, the 8348SOCS3 cells had significantly lower invasion capability than the controls (Figure 4C–4D). Being counted in each group in four different visual fields, the number of migrating cells in the 8348SOCS3 group was significantly lower than that in the 8348plv group (t = 9.590, *P* < 0.05) (Figure 4B).

### The effect of high SOCS3 expression on the invasion and metastasis of CRC *in vivo*

#### Gross observation

**Figure 5:**
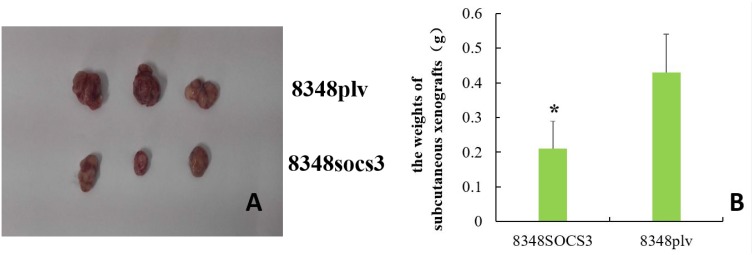
Tumor xenografts in nude mice (**A**) Tumor formation by different cells in nude mice; (**B**) Comparison of the weights of subcutaneous xenografts between 8348SOCS3 group and 8348plv group (^*^P < 0.05).

SOCS3 overexpression inhibited the ability of the 8348 cells to grow as tumor xenografts in nude mice, as shown in Figure 5A–5B. Subcutaneous xenografts in nude mice being weighed 28 days after injection, the tumor weight in the 8348SOCS3 group was significantly lower than that in the 8348plv group (t = 2.802, *P* < 0.05) (Figure 5C).

### Difference in the number of cancer cells in tumor xenografts by HE staining

**Figure 6:**
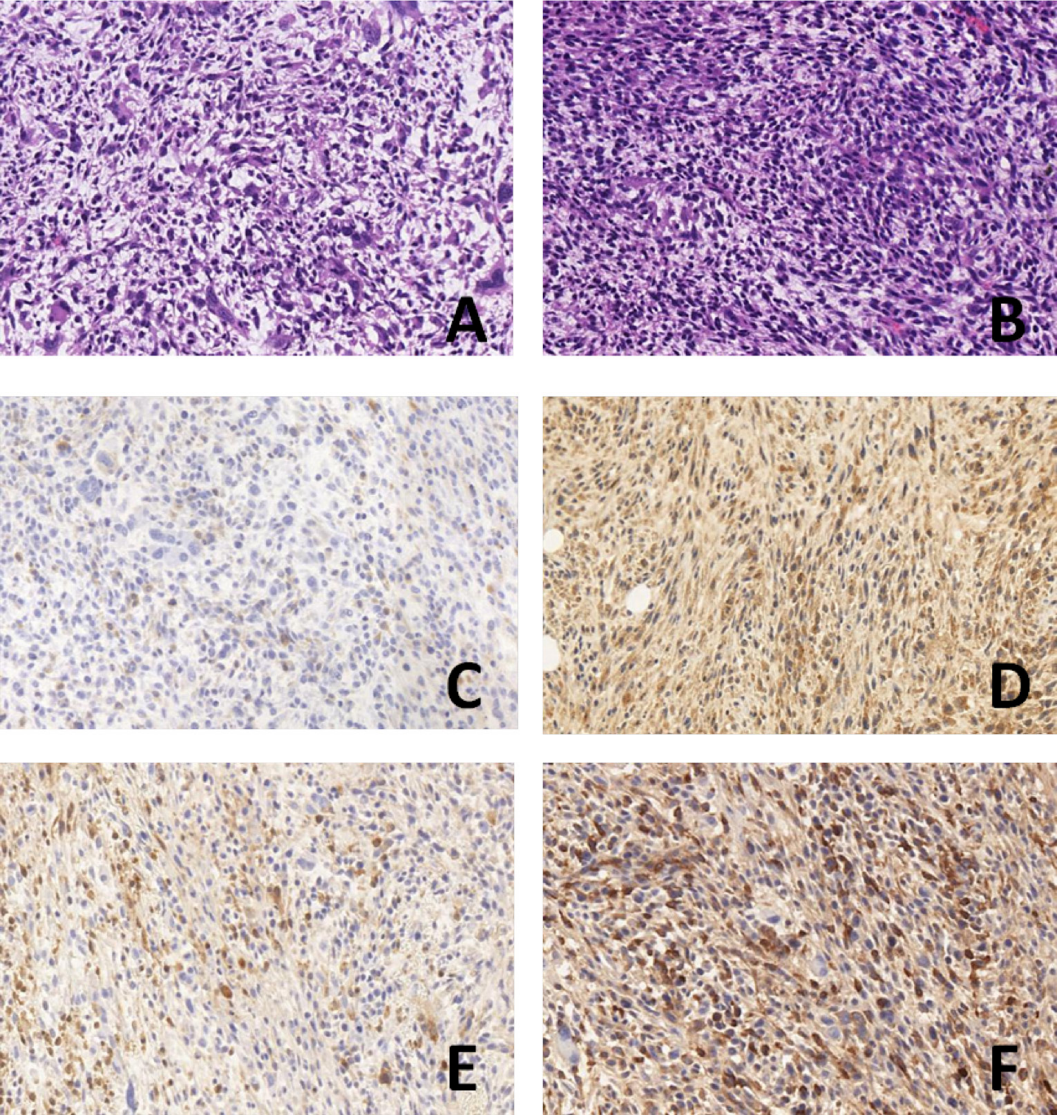
HE and immunohistochemical detection of subcutaneous tumor xenografts in nude mice (× 400) (**A**) 8348SOCS3 group in HE; (**B**) 8348plv group in HE; (**C**) SOCS3 expression in 8348plv group; (**D**) SOCS3 expression 8348SOCS3 group; (**E**) GPRC5A expression in 8348plv group; (**F**) GPRC5A expression in 8348SOCS3 group.

Compared with the 8348plv group, the number of cancer cells was significantly decreased in the 8348SOCS3 group, together with a marked reduction in the number of mitotic Figures and in the degree of tumor necrosis (Figure 6A–B).

#### Expression of SOCS3 and G protein-coupled receptor family C group 5 member A (GPRC5A) in subcutaneous tumor xenografts by immunohistochemical detection

Positive expression of SOCS3 and GPRC5A was mainly located in the cytoplasm. The level of SOCS3 expression was significantly higher in the 8348SOCS3 group (76.8 ± 6.2%) than in the 8348plv group (35.4 ± 4.7%, *P* < 0.05) (Figure 6C–6D). GPRC5A expression was significantly higher in the 8348SOCS3 group (80.2 ± 6.8%) than in the 8348plv group (38.1 ± 5.3%, *P* < 0.05) (Figure 6E–6F). Therefore, GPRC5A might partially mediate its anticancer effect by stabilizing SOCS3 and suppressing the STAT3 signaling pathway.

### Whole-genome expression analysis of the mechanism by which SOCS3 inhibited the growth of CRC cells

#### Effect of SOCS3 overexpression on the expression of relevant genes

Human genome arrays were used to compare gene expression between 8348SCOS3 and 8348plv cells. We found differential expressions of 369 genes in 8348SCOS3 cells versus control cells, among which 193 genes (e.g. *SELENBP1*, *MEGED1*, and *SOX4*) showed significantly increased expression and 176 genes (e.g. *VEGFA*, *SMC4*, and *ABCC5*) showed significantly decreased expression (Table 2).

**Table 2:** Gene expression profile of 8348 SOCS cells

Up regulated gene		Down regulated gene	
Gene name	Ratio	Gene name	Ratio
SELENBP1	2.69	SLC25A3	0.60
MAGED1	2.45	VEGFA	0.60
BASP1	2.44	KLF9	0.60
SPARC	2.41	EFNB2	0.59
FZD2	2.33	CPEB2	0.59
CTGF	2.32	DDX3X	0.60
SOX4	2.29	ABCC5	0.59
FTL	2.28	SMC4	0.59
ATP1B1	2.24	PTBP2	0.59
CSRP2	2.24	UBC	0.58
MCM3	2.24	SLC39A10	0.58
TRIB2	2.23	CCNL1	0.58
CCDC80	2.23	PKM	0.58
NFKBIA	2.22	MAT2A	0.58
GLUD1	2.22	RSRC2	0.57
SCARNA14	2.22	PTBP2	0.56
GRINA	2.21	ZBTB20	0.56
4-Sep	2.21	PNISR	0.53
AP2M1	2.21	MEF2A	0.60
COL1A2	2.21	PHIP	0.61
TMSB10	2.20	UCHL5	0.64
RBFOX2	2.19	GSK3B	0.63
CTNNB1	2.19	TAOK1	0.64
TPM1	2.19	ZBTB44	0.64
GALK1	2.19	RASA1	0.64
SSBP3	2.18	ATRX	0.63
GPX1	2.18	DDX3X	0.63
BAG6	2.17	PPP4R2	0.63
CD248	1.13	ATRX	0.63
CDH5	1.12	GSK3B	0.62

The top 60 up- or down-regulated transcripts of SOCS3-8348 cells

#### Effect of SOCS3 overexpression on signaling pathways

**Figure 7:**
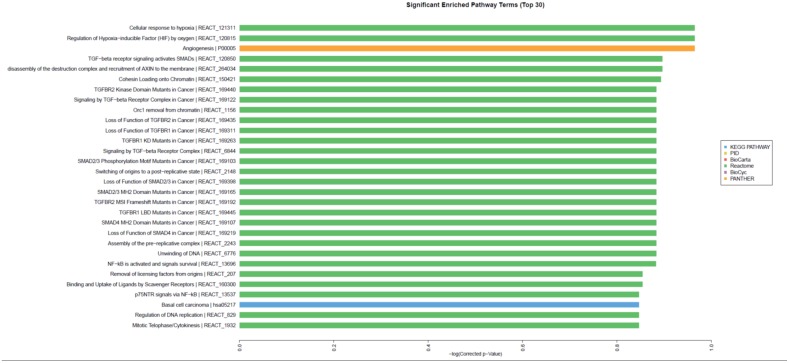
Effect of SOCS3 overexpression on multiple signaling pathways of CRC

**Figure 8:**
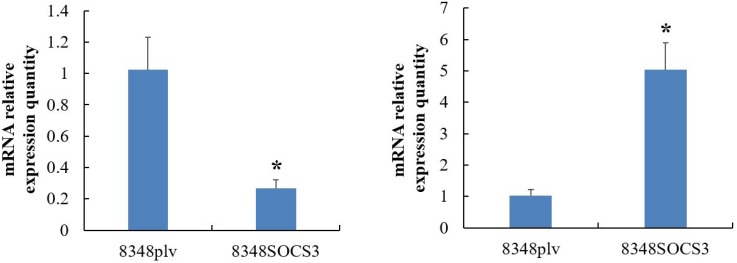
Expression of TGF-β1 and Smad4 in TGF-β/Smads signaling pathway in 8348SCOS3 and 8348plv cells by real-time RT-PCR (**A**) TGF-β1 expression was significantly decreased in 8348S cells; (**B**) Smad4 expression was significantly increased in 8348S cells.

Bioinformatic analysis further demonstrated that high SOCS3 expression might affect multiple signaling pathways in CRC including the TGF-β/Smads signaling pathway, NF-κB pathway, and HIF-MAPK pathway (Figure 7). To investigate the TGF-β/Smads signaling pathway we used qRT-PCR to detect the expression of TGF-β1 and Smad4 in 8348SCOS3 and 8348plv cells and found that TGF-β1 expression was significantly decreased (*P* < 0.05) and Smad4 expression was significantly increased (*P* < 0.05) in 8348SOCS3 cells (Figure 8A–8B).

## DISCUSSION

SOCS3 is a member of the suppressor of cytokine signaling family and also an important inhibitor of the JAK2/STAT3 signaling pathway. The SOCS3 gene is located on human chromosome 17q25.3 and encodes a protein with molecular weight of 24.7 kDa. The SOCS3 protein consists of 225 amino acids that can be divided into an N-terminal domain, a central SH2 domain, and a C-terminal SOCS box. The kinase inhibitory region (KIR) and the central SH2 domain are key domains through which SOCS3 protein exerts its activity. The C-terminal SOCS box functions like an adaptor and can regulate the normal physiological level of SOCS3 inside cells by controlling the degradation of SOCS3 protein. Research has shown that a decrease or loss of SOCS3 expression may be one of the most important causes of malignancies [15]. Gaballah et al. [16] detected SOCS3 expression in highly malignant bladder cancer, low-grade bladder cancer, and normal bladder tissue using RT-PCR and found that SOCS3 expression was significantly lower in highly malignant bladder cancer than in low-grade bladder cancer and normal bladder tissue, suggesting that decreased SOCS3 expression might be associated with the development of bladder cancer. Chen et al. [17] found that the methylation rate of SOCS3 in human endometrial cancer was 88.3%, which was significantly higher than that in complex endometrial hyperplasia (53.3%) and atypical hyperplasia (54.2%). It was speculated that methylation-silenced SOCS3 plays a key role in the pathogenesis of endometrial carcinoma.

To date, few studies have explored the relationship between SOCS3 and the clinicopathological features of CRC, and its mechanisms of action remain unclear. In our current study, we demonstrated that SOCS3 expression was significantly lower in CRC than in normal colorectal tissues by immunohistochemical methods and qRT-PCR, which suggested that decreased SOCS3 expression might be involved in the occurrence and progression of CRC. Further analysis showed that SOCS3 expression was correlated with tumor differentiation degree, depth of invasion, TNM stage, and lymph node metastasis, implying that low SOCS3 expression was associated with other aggressive biological behaviors such as invasion and metastasis. Thus, SOCS3 may be a useful biological indicator for assessing the biological behaviors of CRC and predicting prognosis.

To further study the relationship between SOCS3 level and the biological behaviors of CRC cells such as proliferation, adhesion and invasion, we constructed a CRC cell line that was transfected with SOCS3 vector and showed high expression of SOCS3 (8348SOCS3 cells). Then we compared them with the parental cells transfected with empty vector (8348plv cells). We first examined the effect of SOCS3 overexpression on the growth and proliferation of CRC cells using the CCK-8 method. It was observed that the proliferation rate of 8348SOCS3 cells was remarkably decreased compared with controls. In addition, we applied the transwell method to investigate the effect of SOCS3 overexpression on the invasion capability of CRC cells. It was found that the number of migrating cells in 8348SOCS3 group was significantly smaller than that in 8348plv group after 24 hours of incubation. These results were consistent with the findings of Kathryn et al. [18], which suggested that SOCS3 overexpression could suppress the proliferation and invasion of CRC cells. Therefore artificially inducing SOCS3 expression might provide a new approach to therapy for CRC.

To validate the cytological findings, we performed an *in vivo* experimental study in which 8348SOCS3 cells and 8348plv cells were separately grafted into the subcutaneous tissues of nude mice. After 28 days, the weight of the subcutaneous tumor xenografts in the 8348SOCS3 group was significantly lower than that in the 8348plv group. In addition, compared with the 8348plv group, the number of cancer cells in tumor xenografts was significantly decreased in the 8348SOCS3 group, together with a marked decrease in mitotic figures and in the degree of tumor necrosis. These results suggested that SOCS3 overexpression might inhibit the occurrence and progression of CRC.

GPRC5A, also known as Retinoic acid-induced protein 3 (RAI3), is a retinoic acid-inducible gene. GPRC5A gene locus is at 12p13. LOH of chromosome 12p was found to frequently occur in NSCLCs. It is a newly discovered tumor suppressor gene [19–21]. In GPRC5A-/− mouse tracheal epithelial, the STAT3 signaling was significantly increased [22]. YL Chen et al [22] indicate that the lower SOCS3 level in the GPRC5A-/− cells may be responsible for the persistent activation of STAT3, which regulate cell proliferation, differentiation, survival, invasion and lead to the development of tumors. Using immunohistochemical methods, we showed that GPRC5A expression was significantly higher in subcutaneous tumor xenografts of the 8348SOCS3 group compared with the 8348plv group. We therefore speculated that SOCS3 might at least partially exert its anticancer effect by stabilizing GPRC5A and suppressing the STAT3 signaling pathway.

At present, the mechanism by which SOCS3 influences the occurrence and development of CRC is still not clear. As one of the most potent suppressor proteins in negative regulation of the JAK/STAT signaling pathway, SOCS3 may competitively bind to the phosphorylated Tyr site in the cytoplasmic region of cytokine receptors through its SH2 domain, which mimics the transcription factor STAT, and thus prevent activation of STAT signaling. In addition, SOCS3 can bind to JAK1, JAK2, and TYK2 and directly suppress the catalytic activities of these kinases, thus it can inhibit oncogenes and prevent the occurrence and development of tumors [9, 23]. In the current study, we used a human genome array to compare gene expression between 8348SOCS3 cells and 8348plv cells. We found that the expression of 369 genes was remarkably changed in 8348SOCS3 cells. Of these, 193 genes (e.g., *SELENBP1*, *MEGED1*, and *SOX4*) showed significantly increased expression and 176 genes (e.g., *VEGFA*, *SMC4*, and *ABCC5*) showed significantly decreased expression. Most of these genes were involved in tumorigenesis. So we speculated that SOCS3 might inhibit the occurrence, invasion, and metastasis of CRC by inducing expression of tumor suppressor genes or by suppressing the expression of oncogenes. In addition, bioinformatic analysis further demonstrated that high SOCS3 expression could affect multiple signaling pathways in CRC including the TGF-β/Smads signaling pathway, NF-κB pathway, and HIF-MAPK pathway. The TGF-β/Smads signaling pathway is closely associated with the occurrence and development of tumors in the human body. Abnormality of any of its components can cause a signaling disorder and thus lead to tumorigenesis [24, 25]. By qRT-PCR we showed that TGF-β1 expression was significantly decreased (*P* < 0.05) and Smad4 expression was significantly increased (*P* < 0.05) in 8348SOCS3 cells compared with 8348plv cells. Therefore, SOCS3 may exert its effect by inhibiting the TGF-β1/Smads signaling pathway. TGF-β1, as a fundamental mediator of ECM, plays a critical role in the epithelial-mesenchymal transition (EMT) process by a Smad-dependent pathway. TGF-β1 promotes EMT by which epithelial cells lose their orientation and cell-cell contact, and acquire migratory and invasive properties of cells, resulting in tumor metastasis [26].

In summary, low expression of SOCS3 is closely associated with the occurrence, progression, invasion, metastasis, and other biological behaviors of CRC. Therefore, it may be a useful indicator for assessing the biological behavior of CRC and predicting prognosis. Artificially induced SOCS3 expression may provide a new target for gene therapy of CRC. High SOCS3 expression may exert its antitumor effect by affecting multiple signaling pathways, including the TGF-β/Smads signaling pathway, NF-κB pathway, and HIF-MAPK pathway in CRC. However, its specific mechanisms require further investigation.

## MATERIALS AND METHODS

### Ethics statement

The study was approved by the Clinical Research Ethical Committee of PLA ARMY GENERAL HOSPITAL and informed written consent was obtained from all subjects.

### Detection of SOCS3 expression in CRC

#### Sample collection

Surgically resected CRC specimens were harvested from 40 patients aged 33–70 years (mean: 53 years) who were treated in Department of General Surgery, PLA Army General Hospital from January 1, 2015 to December 30, 2015. Adjacent normal colorectal mucosa was used as the controls. Two blocks of CRC tissue and adjacent normal colorectal mucosa (sized approximately 1.0 × 1.0 × 1.0 cm) were harvested immediately after the surgery. One block was quickly fixed in 10% neutral formaldehyde, and the other was stored at −80°C for qRT-PCR.

### Immunohistochemical determination

The SP (streptavidin perosidase) immunohistochemical method was applied. The rabbit anti-human SOCS3 polyclonal antibody was purchased from Santa Cruz Biotechnology (Santa Cruz, CA, USA; 1:100 dilution). For each slice, five high-power fields were randomly selected for analysis. A comprehensive score was calculated according to the staining intensity and the proportion of positive cells among the total number of tumor cells. [14] Scoring for staining intensity was as follows: 0, no specific staining; 1, light yellow; 2, brownish yellow; and 3, brown. Scoring for the proportion of the positive cells was as follows: 0, less than 5%; 1, 5–25%; 2, 26–50%; and 3, greater than 50%. The final score was obtained by multiplying these two scores; a value of 0–1 was regarded as negative and a score ≥ 2 as positive (+).

### qRT-PCR

Total RNA was isolated from CRC tissue and normal colorectal mucosa tissue with Trizol reagent (Invitrogen Life Technologies, Paisley, UK) according to the manufacturer’s instructions and reverse transcribed. qRT-PCR was performed with Universal SYBR Green PCRMaster Mix (TaKaRa) using specific primers with the following sequences: SOCS3 forward 5′-ATCCTGGTGACATGCTCCTC-3′, reverse 5′-CAAATGTTGCTTCCCCCTTA-3′; β-actin forward 5′-GATCCACATCTGCTGGAAGG-3′, reverse 5′-AAGTGTGACGTTGACATCCG-3′. The reaction conditions were as follows: 94°C for 5 min; 94°C for 30 s, 56°C for 30 s, and 72°C for 40 s (40 cycles); and 72°C for 10 min. Relative quantification was determined using the comparative CT method.

### Effect of SOCS3 overexpression on the biological features of CRC cells

#### Cell culture

8348 cells and 293FT cells were purchased from the Peking Union Medical College Hospital cell bank. The 8348 cells were cultured in RPMI-1640 medium (Sigma, Santa Clara, CA, USA) containing 10% fetal bovine serum (GIBCO BRL, Gaithersburg, MD, USA) and 293FT cells were cultured in DMEM complete medium high-glucose supplemented (Sigma) with 10% fetal bovine serum under standard conditions (37°C, 5% CO_2_, and saturated humidity).

### Cloning of human SOCS3 gene

Total RNA of 8348 cells was extracted by the conventional Trizol method for cDNA synthesis. Cloning of the *SOCS3* gene was performed by a PCR-based method. The PCR product was characterized by 1% agarose gel electrophoresis and subjected to restriction endonuclease digestion. The digestion products were separated by agarose gel electrophoresis and *SOCS3* gene product and vector fragments were separately recovered using a gel recovery kit (Qiagen company). The recovery products were mixed in a molar ratio of 3:1 and incubated with T4 DNA ligase at 16°C overnight. The resultant SOCS3 recombinant plasmid was transferred into *Escherichia coli* DH5α cells for amplification. The transformed bacteria were plated on LB agar plates containing ampicillin and cultured overnight.

### Clone identification

A single colony was inoculated in LB medium containing ampicillin and shaked overnight at 37°C. Plasmids were extracted for screening and identification. The positive bacterial suspension was sent to Sangon Co., Ltd (Shanghai, China) for sequencing. The sequencing results were compared with published databank proteins to confirm the cloned target gene fragments. The suspension containing the correct clone was amplified and recombinant plasmids were extracted and stored at −20°C for further use.

### Packaging of lentivirus

The expression vector for the constructed SOCS3 lentivirus, together with the packaging plasmids and envelope plasmids (Invitrogen), was mixed with lipofectamine 2000 in serum-free high-glucose DMEM medium and incubated at room temperature for 20 min to form the lipofectamine 2000/DNA complex. After trypsin digestion of 293FT cells, approximately 6 × 10^6^ cells were harvested and resuspended in 5 ml of growth medium. After addition of the lipofectamine 2000/DNA complex and thorough mixing, the cells were transferred to a 10-cm cell culture dish containing 5 ml of growth medium and inoculated overnight in a CO_2_ incubator at 37°C. The next day, the medium was changed to complete medium containing 1 mmol/L sodium pyruvate. At 48 and 72 h after transfection, the supernatant was collected and centrifuged at 3,000 rpm for 15 min to remove cell debris before storage at −80°C for further use.

### Cell transfection and flow cytometry

The 8348 cells were collected by centrifugation. Meanwhile, 2 ml of virus supernatant was added to polybrene at a final concentration of 6 mg/L. Cells were resuspended in this solution and incubated in a 96-well plate overnight at a density of 2.5 × 10^5^ cells/ml. The next day, the medium was changed and the cells were cultured for a further 72 h. At 48 and 72 h after transfection, the cell transfection efficiency was observed under a fluorescence microscope. The transfected cells were collected for flow cytometry to obtain 8348 cells with high green fluorescent protein (GFP) expression.

### Detection of SCOC3 expression after transfection by western blotting and qRT-PCR

CRC cells with SOCS3 transfection (8348SOCS3) and without SOCS3 transfection (8348plv) were harvested. Total protein was extracted and the protein concentration was measured with BCA Protein Quantification Kit (Beijing Kangwei Century Biotechnology Co Ltd). Samples were separated by 15% SDS-PAGE and transferred to polyvinylidene difluoride (PVDF) membranes using the semi-dry transfer method. Samples were blocked with 5% skimmed milk powder at room temperature for 2 h, washed with TBST, and incubated with goat anti-human SOC3-3 monoclonal antibody (Santa Cruz Biotechnology) overnight at 4°C. After washing with TBST, HRP-labeled mouse anti-goat second antibody (Santa Cruz Biotechnology) was added and incubated at room temperature for 1 h before a final wash with TBST. Autoradiography was conducted using chemiluminescent substrate. GAPDH protein was used as an internal loading control.

Total RNA was extracted from 8348SOCS3 cells and 8348plv cells by the Trizol method. After reverse transcription, one sample of cDNA was used for a gradient dilution for generation of a standard curve; PCR was performed simultaneously with the other samples. The standard curve was drawn for relative quantitative analysis.

### Effect of SOCS3 overexpression on the growth and proliferation of CRC cells using the CCK-8 method

8348SOCS3 cells and 8348plv cells were harvested and rinsed with 5 ml of PBS. Five hundred microliters of trypsin was added to digest the cells, and the digestion was terminated by addition of DMEM with 10% FBS. Cells were counted and plated in a 96-well plate at a density of 2 × 10^4^ cells/ml. Four parallel wells were set up with 100 μl cellular suspension in each well, and the plate was placed in a CO_2_ incubator and incubated overnight. At fixed measurement time points 10 μl of CCK-8 agent was added to every well, followed by incubation in a 5% CO_2_ incubator at 37°C for 2 h. The optical density (OD) of the cells was measured with a detection wavelength of 450 nm. The OD value on the first day was used as the blank control.

### Effect of SOCS3 overexpression on the invasion of CRC cells using a transwell method

Matrigel was added to the upper chamber of the transwell, which was then incubated at 37°C for 30–40 min for gelling. The 8348SOCS3 and 8348plv cell suspensions were digested and incubated in the upper chamber of the transwell at a density of 5 × 10^4^ cells/ml for 24 h. After fixation in 4% polyformaldehyde for 15–20 min the cells were observed and photographed under a fluorescence microscope.

### Effect of high SOCS3 expression on the invasion and metastasis of CRC *in vivo*

#### Gross examination

The 8348SOCS3 cells and 8348plv cells were collected by trypsin digestion. With three nude mice in each group, each mouse was designed to receive 1 × 10^7^ cells in a 100-μl volume at each of the injection sites. The 3% pentobarbital sodium was diluted 1:10 with PBS before use. The dose of the anesthetic (after dilution) was 20 μL/g for each mouse. Cells were grafted under the dorsal skin of nude mice. After 28 days, the tumor sizes were measured and photographed.

### Detection of SOCS3 and GPRC5A expression by HE staining and immunohistochemistry

The tumor tissue was cut into tissue blocks sized 0.5 cm^3^ to produce paraffin-embedded tumor sections. The other steps for immunohistochemical determination were performed as described above. Using known SOCS3- and GPRC5A-positive sections as the positive controls, cells with intracellular brown-yellow particles were regarded as positive. A total of 100 cells were counted in each high-power field (× 400). The rate of positive cells was calculated as number of positive cells/total number of cells × 100%.

### Whole-genome expression analysis of the mechanism by which SOCS3 regulates the growth of CRC cells

Total RNA was extracted from 8348SOCS3 cells and 8348plv cells using Trizol reagent. Genome expression analysis was performed using an Illumina Human HT-12 v4 BeadChip (Illumina, San Diego, CA, USA) at the Beijing Qian Zhao Xing Ye Biological Technology Co., Ltd. (Beijing, China). The beadchips were scanned on the Illumina Bead Array 500GX Reader using Illumina BeadScan image data acquisition software. Illumina BeadStudio software was used for preliminary data analysis. The preliminary data were normalized using sample averages; the sample intensities were scaled by a factor equal to the ratio of average intensity of a sample to the average intensity of the given sample [8]. 8348SOCS3 cells and 8348plv cells were regarded as the given sample. Each sample was repeated three times. An Illumina custom algorithm was used to compare 8348SOCS3 cells with 8348plv cells. A difference score for a probe (diff score) indicates differential gene expression between the two groups. For each gene, the diff scores of corresponding probes were averaged. The results were validated using qRT-PCR.

### Statistical analysis

All data analysis was performed using SPSS 13.0 software (SPSS Inc., Chicago, IL, USA). Measurement data were presented as means ± SD and analyzed using Student’s t test. Comparison of count data was performed using chi square test. *P* < 0.05 was considered statistically significant.
